# Dietary Fats, Human Nutrition and the Environment: Balance and Sustainability

**DOI:** 10.3389/fnut.2022.878644

**Published:** 2022-04-25

**Authors:** Erik Meijaard, Jesse F. Abrams, Joanne L. Slavin, Douglas Sheil

**Affiliations:** ^1^Borneo Futures, Bandar Seri Begawan, Brunei; ^2^Department of Ecology, Charles University in Prague, Prague, Czechia; ^3^School of Anthropology and Conservation, Durrell Institute of Conservation and Ecology (DICE), University of Kent, Canterbury, United Kingdom; ^4^Global Systems Institute, Institute for Data Science and Artificial Intelligence, University of Exeter, Exeter, United Kingdom; ^5^Department of Food Science and Nutrition, University of Minnesota, St. Paul, MN, United States; ^6^Forest Ecology and Forest Management Group, Wageningen University and Research, Wageningen, Netherlands

**Keywords:** nutrition, dietary fat, environment – agriculture, sustainable development goals, planetary boundaries, undernourishment

## Abstract

Dietary fats are essential ingredients of a healthy diet. Their production, however, impacts the environment and its capacity to sustain us. Growing knowledge across multiple disciplines improves our understanding of links between food, health and sustainability, but increases apparent complexity. Whereas past dietary guidelines placed limits on total fat intake especially saturated fats, recent studies indicate more complex links with health. Guidelines differ between regions of general poverty and malnutrition and those where obesity is a growing problem. Optimization of production to benefit health and environmental outcomes is hindered by limited data and shared societal goals. We lack a detailed overview of where fats are being produced, and their environmental impacts. Furthermore, the yields of different crops, for producing oils or feeding animals, and the associated land needs for meeting oil demands, differ greatly. To illuminate these matters, we review current discourse about the nutritional aspects of edible fats, summarize the inferred environmental implications of their production and identify knowledge gaps.

## Introduction

Our ancestors have been dubbed “fat hunters” (1), indicating our needs for dietary fats. Oils and fats (further referred to as “fats”) are an important component of our diets and, while in parts of the world there is overconsumption (2), an estimated 720 to 811 million people were undernourished worldwide in 2020 (3). About 25–30% of daily energy needs in a normal modern healthy diet comes from fats (4), and facilitating access of undernourished people to fats is important. Currently, an estimated 45 million tons (Mt) of dietary fat per year are required to reach recommended levels of fat consumption (5). This includes both a reduction of fat consumption in regions of overconsumption of especially animal fats, and an increase in areas of underconsumption. If this “fat gap” is projected to 2050, an additional 88–139 Mt are required. This will mostly come from soybean oil, which in 2019, globally contributed 9.88 g of fat per capita per day, palm oil 7.17, sunflower oil 4.35, butter and ghee 3.62, and rapeseed and mustard oil 3.51 (6). Combined these five sources contribute an estimated 62% of the global consumption. Nearly 80% of the fats produced for human consumption are derived from oil crops, for which global production is currently around 208 Mt of oil (7). The remaining fat production derives from animal fats (“dairy” which includes butter, ghee, cheese, milk etc.), which was 46 Mt in 2019 (8) with additional animal fats produced in lard (6 Mt) and tallow (7.3 Mt) (6).

The health aspects of fats as well as the environmental impacts of their production receive significant media attention. How to best produce oils from plants, and fats from animals feeding on plants, is therefore not only of nutritional and human health importance but also relates to planetary health. For example, different oil crops have different yields and land requirements to produce the same amount of oil (9, 10). These crops are also grown in different parts of the world, with oil palm a typical crop of the tropics, and soybean of the subtropics and temperate climate zones. For many uses, oils are interchangeable (11), so a reduction in the production of one type of vegetable oil will result in an increase in another, and thus affect where land is allocated to oil production.

Here we review current knowledge of the nutritional and health aspects of dietary fats, and how this affects people in different parts of the world. We also review how the demand for dietary fats could be met and what the possible environmental consequences will be.

## Affordable Fats of Satisfactory Nutritional Value

Fats (or lipids) are the primary structural components of cellular membranes and are also sources of energy (12). Furthermore, fats provide essential fatty acids and facilitate the absorption of fat-soluble vitamins (A, D, E, and K) (13). Fats include various food oils—with “oils” being colloquially used for fats that remain liquid at room temperature. Dietary fats fall into three categories based on the number of chemical double bonds: monounsaturated fats, polyunsaturated fats, and saturated fats. A fourth category, trans fats, forms during partial hydrogenation of vegetable oils produced industrially (e.g., margarines), or naturally in beef, lamb, and dairy products (5). Monounsaturated fats are mostly found in fish, many plant-derived oils, nuts, and seeds. Saturated fats primarily occur in animal products and in palm oil (12). In practice, such general terms can seldom be used with precision though. Most fats are complex chemical mixtures of all major saturated fatty acids in differing proportions, along with many other fatty and non-fatty acids (14). All these different types of fats have different impacts on health, sometimes negative, or sometimes positive, such as in the case of polyunsaturated essential fatty acids. The science around the complex interactions between hundreds of thousands of food components, how these interact among each other, and how they interact in turn with different components of human health, is still developing (15).

While fats are one of the potential sources of energy in humans, some fats are essential. One of these is alpha-linolenic acid, an omega-3 fatty acid, that is particularly abundant in walnuts, rapeseed, some legumes, flaxseed, and green leafy vegetables (16). Another essential fat is linoleic acid, an omega-6 fatty acid, which plays an important role in functions such as cell physiology, immunity, and reproduction. Linoleic acid is an important component of breast milk, and, in many vegetable oils, it represents more than 50% of the lipid content. High amounts of linoleic acid are also present in nuts, cereals, legumes, some meats, eggs, and dairy products (17). Fats also influence bioavailability of fat-soluble vitamins, with a Western diet high in fat causing alterations of gut microbiota and potentially reducing the bioavailability and function, and possibly introducing potential toxicities, of these vitamins (18). Finally, pentadecanoic acid and heptadecanoic acid, found mainly in milk and other dairy products, are trace saturated fatty acids which cannot be synthesized by the human body in sufficient amounts and have therefore been proposed as essential in small doses (19).

Dietary guidance around the world has evolved into desirable dietary patterns. Recommendations now support food practices, such as the Mediterranean diet, which are often high in dietary fat, but include other recommended foods, such as vegetables, fruits, legumes, and whole grains (20). These dietary patterns have implications beyond cardiovascular disease with new emphasis on brain health, gut health, and weight management. Additionally, a diet’s fat quality is recognized as more important than the saturated fat content (14, 21). A meta-analysis by Astrup et al. (14) indicated that replacement of fat with carbohydrate was not associated with lower risk of coronary heart disease, and may even be associated with increased total mortality. Also, systematic studies find no significant association between saturated fat intake and coronary artery disease or mortality, and some even suggested a lower risk of stroke with higher consumption of saturated fat (14). High fat diets, even those high in saturated fat, may be protective in cardiometabolic disease as when fats are removed from the diet they are replaced by carbohydrates which are linked to health risks (22). In the context of contemporary diets, therefore, these observations would suggest there is little need to further limit the intakes of total or saturated fat for most populations (14). Similar changes surround past concerns around cholesterol and heart disease. Cholesterol – mostly found in animal fats – is essential for human life but also not a required nutrient as, if it isn’t ingested, the human body can make what it needs. It is a component of the cell membrane, a precursor molecule in the synthesis of vitamin D, steroid hormones, and sex hormones, and also plays a role in the absorption of fat-soluble vitamins (23). The effects of dietary fats on cardio-vascular disease risk have traditionally been estimated from their effects on serum cholesterol, although the thinking about health implications of these measures are changing (14, 24). Also, there is ongoing debate about the optimal intake ratios of various omega-3, 6, and 9 fatty acids (25–27).

**FIGURE 1:**
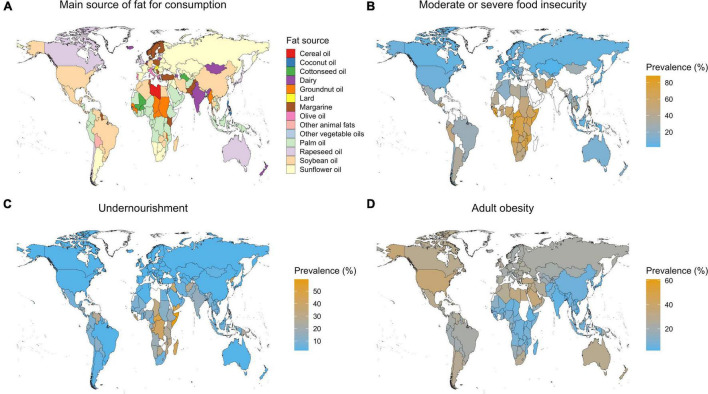
The dominant sources of fat consumption **(A)**, and the prevalence of severe food insecurity **(B)**, undernourishment **(C)**, and adult obesity **(D)** per country. For details on data and methods used for maps refer to Supplementary Materials.

Most of the nutritional and health studies have evaluated the role of different fats on people in the global North, often in relation to the 1.9 billion adults worldwide that are overweight (28). Fat limitation in early dietary guidance primarily applied to obese societies because fats contain 9 kcal/g vs. 4 kcal/g for carbohydrates and protein, but for people who are underweight energy-dense food is important. Geographically undernourishment and food insecurity are concentrated in sub-Saharan Africa, parts of Asia and the Caribbean (Figure 1). The “depth of the food deficit,” i.e., a measure providing an estimate of the number of additional calories the average individual needs is especially high in countries such as Haiti (530 kcal/person/day), or the Central African Republic (380 kcal kcal/person/day) (29). Countries with high food deficits coincide with parts of the world with large fat gaps: Eastern, Northern, Middle and Western Africa; East, Southeast and South Asia, and the Caribbean (5). Understanding fats in diets of undernourished people is important, and may also impact infants through quality of breast milk related to fat intake by mothers (30). Regional studies in South America note, however, that feeding energy-rich micronutrient-poor foods to undernourished people can promote obesity (31). The extent to which fats can contribute to closing the food deficit without resulting in obesity (32, 33) remains therefore unclear, although dietary fats will likely play some role in increase energy intake among undernourished people.

Addressing the food deficit requires affordability and availability (34). Compared to other food groups, fats are cheap. With a cost per person per day of less than USD 0.20, fats contribute about 4% to the average global cost of a healthy diet. In comparison, the cost is ca. USD 0.40 for starch staples, USD 0.60-1.00 for protein-rich foods, and around USD 0.70 for dairy, fruits and vegetables (34). Compare these costs to the international poverty line for low-income countries of USD 1.90/day (35). Fat prices vary with type and origin (36), but generally affordability favors local productions. Transport and logistics costs in tropical America and the Caribbean, for example, made up 20% of the cost of food products (37). Figure 1 shows that crops such as peanut in central Africa and palm oil central Africa and in South-East Asia could play important roles in the local supply of affordable fats.

## Comparing Oils and Fats in an Environmental Context

### Environmental Impacts of Fat Production

Expanding agriculture is the principal cause of biodiversity decline (38), a major contributor to nitrogen and phosphorus pollution (39), to land degradation (40) and to freshwater depletion (41). From 2003 to 2019, global cropland areas increased by 9%, with a near doubling of the annual expansion rate, primarily due to agricultural expansion in Africa and South America (42). Half of the new cropland area (49%) replaced natural vegetation and tree cover, indicating a conflict between the goals of producing food and protecting terrestrial ecosystems (42). Such expansion risks lasting damage to the habitability of the planet (43). With 331 million ha, oil crops make up about 23% of the total land area allocated to crop production, but at 41% the oil crops area expanded even more than overall croplands between 2003 and 2019 (6).

**FIGURE 2:**
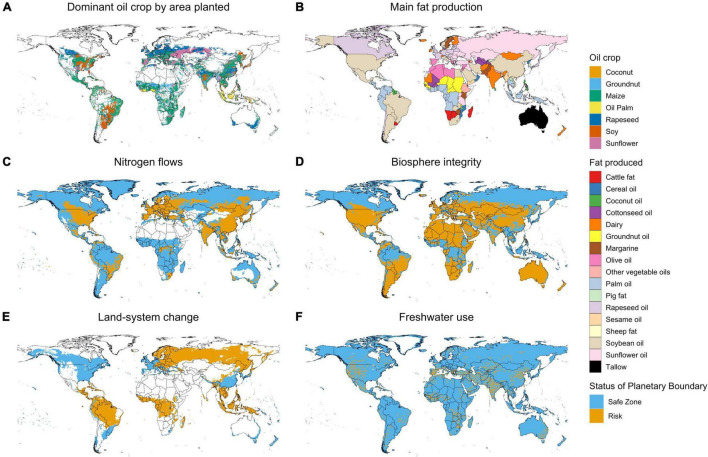
**(A–F)** The status of planetary boundaries for nitrogen flows, biosphere integrity, land-system change and freshwater use compared to the main oil crop and fat production areas. For details on data and methods used for maps refer to Supplementary Materials.

In relation to the production of fats, excessive nitrogen flows are focused on areas favoring production of soybean and dairy (Figures 2A–C). Concerns about biosphere integrity coincide with areas producing soybean, sunflower, dairy, olive oil, and groundnut (Figures 2A,B,D). Concerns about land-system change dominate the wet tropics where oil palm and soybean are grown and parts of the palearctic where sunflower and tallow are produced (Figures 2B,E). Concerns around freshwater focus on drier areas in the western United States, Mediterranean, and South Asia where olive, sunflower, dairy and soybean predominate (Figures 2A,B,F).

### Meeting Future Fat Demands

With an increasing global population, demand for fat will increase and this will mostly be met through oil crops. Because of the energetic costs of transforming plant material into animal material, more land is needed to produce a certain amount of animal fat than directly from plants. Animals do not only eat oil crops, but also consume plants such as grasses not normally eaten by people. There is debate surrounding the extent and conditions under which production of animal fats and vegetable oils compete, with much depending on the particular production systems compared (44).

An increase in fat production through oil crops can be achieved in two ways: (1) by increasing the yield of existing crops, thus producing more oil on the same amount of land, or (2) by allocating new land for the production of oil (9). Currently just four crops—oil palm, soybean, sunflower and rapeseed—provide most vegetable oil. Though values can vary considerably with context, typical yields differ among crops. For example, oil palm typically yields 2.84 tons of oil per ha, while soybean produces 0.45 tons, and groundnut 0.18 tons (10). As a result oil palm supplies 36% of global vegetable oil volumes on just 8.6% of the land allocated to oil production (10). Comparable figures for soybean are 25.5% of production on 39% of land, for rapeseed and mustard 11.3% on 12%, and for sunflower 9% on 8.3% (10).

The impacts of oil crop expansion on natural ecosystems is extensive in, for example, South-East Asia, where oil palm and coconut replaced tropical forest (45, 46), South America, where soybean has replaced tropical forest and savannahs (47), and equatorial Africa where maize, groundnut and cotton are expanding into tropical forest and savannah (48). Expansion of oil crops also impacts Australia where the area of rapeseed has increased 100-fold over the past 40 years (6), including in areas of threatened natural ecosystems (49), and the United States, where soybean and maize has expanded into large areas of relatively biodiverse natural grass and scrublands in recent decades (50). Similar processes have occurred with rapeseed and sunflower cultivation in Russia, Kazakhstan and Ukraine. Quantifying such impacts remains imprecise, because, except for oil palm (51), none of these crops have been globally mapped at sufficient resolution.

## Discussion

Our review of the impact of fat production and consumption on human and planetary health indicates potential tradeoffs and synergies from different fat choices. Fat demand is likely to increase to feed an increasing number of people. In parts of the world with widespread overweight, reduced fat intake and more balanced consumption of different and essential fats is needed. In parts of the world with high incidence of undernourishment, increased production of local, affordable fats seems important, although global recommendations still call for avoidance of fat and especially saturated fat (3). The availability of products such as Plumpy’Nut, a peanut-based paste that consists for one-third of fat and is used for treatment of severe acute malnutrition, indicates the importance of fats in regions of undernourishment. Better guidance is needed regarding which fats might help address undernourishment, without adverse health impacts, and costs.

While the health impacts from consumption of saturated fats may have been overstated, dairy has a high environmental footprint, and use should be reduced. Increased consumption of lard and tallow proportionally to pork and beef production, on the other hand, would allow more optimal use of edible fat (5). Nevertheless, in terms of planetary health, the production of plant-based fats has lower negative impacts than the production of animal fats, and growing crops with high oil yields is recommended as this spares land. We must moderate the impacts from crop expansion on biodiversity and natural ecosystems and depletion of groundwater (39). How to best seek a balance between these different objectives is difficult to determine because ultimately many choices are value driven – e.g., saving orangutans from oil palm expansion versus the need to provide poor people with affordable fats. Nevertheless, some general patterns can guide decision-making on future fat production choices.

Palm oil is an important oil for cultural and price reasons in large parts of South-East Asia and central Africa, and its alleged negative health impacts because of high saturated fat content is increasingly questioned (52, 53). Among the oil crops it is the most land-efficient fat and efficiency could be further improved, especially through mechanized harvesting and better chemical management, but deforestation must be avoided to protect biodiversity and carbon stocks (9). Peanut provides a healthy and cheap source of oil, and improved peanut production could reduce fat gaps in key regions of human population growth (i.e., Africa and south Asia). Because both palm oil and peanut oil are relatively cheap, they will remain important oils for many people. Coconut, another oil crop of tropical regions is an important source of fat to many people. Impacts on health remain debated (54), and differ for different types of coconut oil (55). Furthermore, there are concerns about coconut’s environmental impacts, especially on tropical islands with high species endemism where loss of natural ecosystems because of coconut expansion threatens biodiversity (46).

Soybean oil, as the largest oil crop in area, will likely remain a leading source of oil, and it is also a key component of animal feed. Reducing pork and poultry production can lead to reduction in soybean oil production and spare land in regions of high deforestation such as South America. There are concerns about negative health impacts related to the lipid profile of sunflower oil, especially its very high omega-6 to omega-3 ratio (5), but it is difficult to generalize about this, also because there are different types of sunflower oil that vary significantly in their oleic, linoleic (omega-6) and stearic acid content.

Finally, further research is needed in the opportunities to produce fats at scale from microbial and insect sources. Algal, yeast and other microbial oils have major potential for the production of design oils that meet human health requirements, but remain relatively expensive to produce (56). The environmental impacts of such oils depend on the need for a feedstock, with especially carbon-based feedstocks (often sugars) requiring crop land for their production (57). Edible insects are an alternative fat and protein source with low greenhouse gas emissions and low land use, and with at least 2000 edible species of insects (58) there is much to choose from. Given these developments, it is likely that dietary guidance on fats will continue to emerge and change with developments in science, technology and future challenges and opportunities.

## Author Contributions

EM conceived the initial study, conducted literature review, wrote the initial manuscript, and coordinated the study. JA implemented the mapping for this study and contributed to the manuscript revisions. JS provided input on the text on nutrition. DS provided input into the original study design and edited the manuscript on several occasions. All authors contributed to the article and approved the submitted version.

## Conflict of Interest

EM is employed by company Borneo Futures. EM declares a potential conflict of interest through paid work he has done regarding research on oil crops, including on oil palm, coconut, rapeseed, soybean, and sunflower. The remaining authors declare that the research was conducted in the absence of any commercial or financial relationships that could be construed as a potential conflict of interest.

## Publisher’s Note

All claims expressed in this article are solely those of the authors and do not necessarily represent those of their affiliated organizations, or those of the publisher, the editors and the reviewers. Any product that may be evaluated in this article, or claim that may be made by its manufacturer, is not guaranteed or endorsed by the publisher.
