# Supplementary material for: Dietary Fats, Human Nutrition and the Environment: Balance and Sustainability
Source: Front Nutr. 2022 Apr 25;9:878644. doi: 10.3389/fnut.2022.878644 (PMC9083822; doi:10.3389/fnut.2022.878644)
Supplement: Supplementary file 1 [file Data_Sheet_1.docx]

Supplementary Material

# Supplementary Methods and Data sources

To explore the impact of food fat sources (oils and fats) on human health and the environment we considered the spatial distribution of dietary fats alongside those of the Sustainable Development Goals (SDGs) and the Planetary Boundaries (PB). To do so, we compiled estimates of fat consumption and production globally and compared to various indicators for SDG 2 (Global goal to end hunger by 2030) and the PBs.

***Fat production and consumption***

The main source of food fats varies around the world. In most countries, production and consumption are aligned, but this is not always the case. The main food fats produced and consumed per country is derived from the FAO Supply Utilization Accounts ([https://www.fao.org/faostat/en/#data/SCL](about:blank)) data for the year 2019. All items in the aggregated item lists “Oils and Fats” for the elements “Production Quantity” and “Fat supply quantity” were used as source data for production and consumption, respectively. The data was used as downloaded from the FAOSTAT website, however we performed some category aggregation: all “Butter” and “Ghee” items were merged into a “Dairy” category, “Maize oil” and “Rice bran oil” were aggregated to “Cereal Oil”, and multiple items for “olive oil” and “palm oil” were aggregated to one item for each. The choropleth maps for consumption and production in Figures 1 and 2, respectively, display the category with the highest value for each country.

***Oil crop distribution***

The spatially explicit oil crop distribution data was downloaded from the Spatial Production Allocation Model (SPAM) 2010 dataset for global spatially disaggregated crop production statistics (International Food Policy Research Institute, 2019; Yu et al., *under review*). The physical area of coconut, sunflower, maize, oil palm, rapeseed, groundnut, and soybean were downloaded as GeoTIFF files at a 10 x 10 km grid-cell resolution. The crop areas per raster cell were compared and the dominant crop in each raster cell was determined.

***Sustainable Development Goals (SDG)***

Food fat supply and consumption play an important role in achieving the goal of SDG 2 - "End hunger, achieve food security and improved nutrition, and promote sustainable agriculture”. We downloaded data from the FAO Suite of Food Security Indicators ([https://www.fao.org/faostat/en/#data/FS](about:blank)). We analyzed three datasets that are used as indicators for SDG 2.1 and SDG 2.2:

- Prevalence of obesity in the adult population (18 years and older).
- Prevalence of undernourishment, 3-year averages.
- Prevalence of severe food insecurity in the total population. This is indicator 2.1.2 in the SDG framework, to monitor target 2.1 ("By 2030, end hunger and ensure access by all people, […], to safe, nutritious and sufficient food all year round").

We downloaded all data for the most recent year available for each indicator. We then plotted the value for each country.

***Planetary Boundaries (PB)***

We considered the impact of food fat production on important environmental processes and quantities. To explore this, we adopted the PB concept, considering four PBs that are strongly influenced by agricultural activity: biosphere integrity, land-system change, freshwater use, and nitrogen flows. We plotted spatially explicit maps of the current status of PBs following the methods of Gerten *et al.* (2020). For full details on the methodology please refer to Gerten *et al.* (2020). We received the data upon request to the authors and modified their approach by considering only two categories for the PBs (instead of their three categories), “Safe zone” and “Risk”. The “Safe zone” is any area that is below the PB and the “Risk” areas are any areas that are beyond the PB threshold for the respective PB. The threshold of the PB for biosphere integrity is set at a level of 90%. The PB for land-system change is set at 50% for temperate forest biomes and 85% for boreal and tropical forest. The PB threshold for freshwater is determined by a maximum monthly withdrawal as a percentage of mean monthly river flow; for low-flow months: 25%; for intermediate-flow months: 30%; for high-flow months: 55%. The PB threshold for N flows limits leached N concentrations in surface waters to 1 mg l^–1^ of N.

**References**

Gerten, D., Heck, V., Jägermeyr, J., Bodirsky, B. L., Fetzer, I., Jalava, M., ... & Schellnhuber, H. J. (2020). Feeding ten billion people is possible within four terrestrial planetary boundaries. *Nature Sustainability*, *3*(3), 200-208.

International Food Policy Research Institute, 2019, "Global Spatially-Disaggregated Crop Production Statistics Data for 2010 Version 2.0", [https://doi.org/10.7910/DVN/PRFF8V](about:blank), Harvard Dataverse, V4

Yu, Q., You, L., Wood-Sichra, U., Ru, Y., Joglekar, A. K. B., Fritz, S., Xiong, W., Lu, M., Wu, W., and Yang, P.: A cultivated planet in 2010: 2. the global gridded agricultural production maps, Earth Syst. Sci. Data Discuss., https://doi.org/10.5194/essd-2020-11, in review, 2020. doi: 10.5194/essd-2020-11
